# Tai Chi practice enables prefrontal cortex bilateral activation and gait performance prioritization during dual-task negotiating obstacle in older adults

**DOI:** 10.3389/fnagi.2022.1000427

**Published:** 2022-11-18

**Authors:** Yan Chen, Aiying Wan, Min Mao, Wei Sun, Qipeng Song, Dewei Mao

**Affiliations:** ^1^College of Sport and Health, Shandong Sport University, Jinan, Shandong, China; ^2^School of Kinesiology, Shanghai University of Sport, Shanghai, China; ^3^School of Nursing and Rehabilitation, Cheeloo College of Medicine, Shandong University, Jinan, Shandong, China

**Keywords:** Tai Chi, functional near-infrared spectroscopy, dual-task, negotiating obstacle, older adult, prefrontal cortex

## Abstract

**Background:**

With aging, the cognitive function of the prefrontal cortex (PFC) declined, postural control weakened, and fall risk increased. As a mind–body exercise, regular Tai Chi practice could improve postural control and effectively prevent falls; however, underlying brain mechanisms remained unclear, which were shed light on by analyzing the effect of Tai Chi on the PFC in older adults by means of functional near-infrared spectroscopy (fNIRS).

**Methods:**

36 healthy older adults without Tai Chi experience were divided randomly into Tai Chi group and Control group. The experiment was conducted four times per week for 16 weeks; 27 participants remained and completed the experiment. Negotiating obstacle task (NOT) and negotiating obstacle with cognitive task (NOCT) were performed pre- and post-intervention, and Brodmann area 10 (BA10) was detected using fNIRS for hemodynamic response. A three-dimensional motion capture system measured walking speed.

**Results:**

After intervention in the Tai Chi group under NOCT, the HbO_2_ concentration change value (ΔHbO_2_) in BA10 was significantly greater (right BA10: *p* = 0.002, left BA10: *p* = 0.001), walking speed was significantly faster (*p* = 0.040), and dual-task cost was significantly lower than pre-intervention (*p* = 0.047). ΔHbO_2_ in BA10 under NOCT was negatively correlated with dual-task cost (right BA10: *r* = −0.443, *p* = 0.021, left BA10: *r* = −0.448, *p* = 0.019). There were strong negative correlations between ΔHbO_2_ and ΔHbR under NOCT either pre-intervention (left PFC *r* = −0.841, *p* < 0.001; right PFC *r* = −0.795, *p* < 0.001) or post-intervention (left PFC *r* = −0.842, *p* < 0.001; right PFC *r* = −0.744, *p* < 0.001).

**Conclusion:**

Tai Chi practice might increase the cognitive resources in older adults through the PFC bilateral activation to prioritize gait performance during negotiating obstacles under a dual-task condition.

## Introduction

Cognitive function (Zhang et al., 2018; Chen et al., 2020; Demurtas et al., 2020; Jung et al., 2020; Yue et al., 2020a, b; Cheval et al., 2022) and postural control/stability (Wang et al., 2016; Zou et al., 2019a; Koohsari et al., 2020; Song et al., 2021; Veronese et al., 2021; Zhang et al., 2021; Cunha et al., 2022) are simultaneously declining with aging. In daily life, walking across obstacles and/or walking under dual-task conditions require greater cognitive function (Holtzer et al., 2014; Chen et al., 2017; Zhang et al., 2020; Lu et al., 2021) and fine postural control (Harley et al., 2009). Performing dual task or multi-task like talking over the phone while walking is regularly needed, but is very complex and challenging as it requires cognition-balance integration that are naturally degenerating among older adults (Camicioli et al., 1997; Lundin-Olsson et al., 1997; Hausdorff et al., 2008; Schwenk et al., 2010). The above-mentioned scenario seems to be supported by the capacity-sharing theory (Tombu and Jolicoeur, 2003; Leone et al., 2017; Kunstler et al., 2018), suggesting that single-task and/or dual-task is damaged if cognitive demand exceeds the total capacity of available resources. Failure in negotiating obstacles was reported to associate with the increased risk for age-related falls (Toba et al., 2005), which resulted in negative consequences including bruises, sprains, cerebral trauma, joint dislocation, fractures and even death (Konak et al., 2016).

Cognitive impairment has been identified as an important cause of falls in older adults (Sheridan and Hausdorff, 2007; Verghese et al., 2012), and executive function was closely related to falls, disability and death (Sheridan and Hausdorff, 2007; Kearney et al., 2012; Montero-Odasso et al., 2012; Yu et al., 2021). Executive function referred to the advanced cognitive control process for attaining a specific goal (Moriguchi and Hiraki, 2013). Dual-task and negotiating obstacle tasks (NOTs) were related to different aspects of executive function, i.e., negotiating obstacle related to motor planning and visuospatial ability, whereas dual-task related to attentional allocation and working memory (Galna et al., 2009; Herman et al., 2010; Beurskens and Bock, 2012; Beurskens et al., 2014). Executive function was vulnerable to special population like older adults (West, 1996; Raz et al., 1998; Buckner, 2004; Head et al., 2004; Hedden and Gabrieli, 2004; Gu et al., 2019; Zheng et al., 2021) and other mental or physical disabilities (Zou et al., 2018; Cai et al., 2020; Kong et al., 2022; Sun et al., 2022).

A strong link between walking and cognition, especially the high-order executive function, has been observed (Tinetti et al., 1988; Yogev-Seligmann et al., 2008; Pieruccini-Faria et al., 2019). Several imageology studies have indicated that the prefrontal cortex (PFC) was primarily responsible for executive function during walking and its associated brain network like frontoparietal network was activated (Garavan et al., 2002; Amboni et al., 2013; Holtzer et al., 2014). Of note, the frontal lobe was one of the most sensitive brain structures linked to aging (Hedden and Gabrieli, 2004)and its Brodmann area 10 (BA10) was reported to specifically associate with executive function (Semendeferi et al., 2001), such as memory and recall of information, problem-solving (Szczepanski and Knight, 2014), participation in memory extraction during task execution and complex reasoning tasks (Braver and Bongiolatti, 2002; Roca et al., 2011; Dong et al., 2019). Therefore, BA10 was chosen as the region of interest (ROI) in this study.

The portable functional near-infrared spectroscopy (fNIRS) is feasible in assessing functional brain activities during walking (Atsumori et al., 2010; Nieuwhof et al., 2016; Balardin et al., 2017; Pinti et al., 2018). FNIRS is an imaging technique to measure cortical hemodynamic response (Herold et al., 2018; Yu et al., 2021, 2022) and analyze local cortical activation based on the neurovascular coupling mechanism (Obrig and Villringer, 2003; Di Lorenzo et al., 2019). FNIRS is practically avoids motion artifacts, and is suitable for cortical activity assessment when older adults walk in a real-world environment.

As a mind–body exercise, Tai Chi has gained greatest popularity among aging population due to its associated health benefits (Zou et al., 2021) generating from long-term practice. Taking closer look, Tai Chi has great emphasis on weight shifting during dynamic walking and its eye-body (upper-limb and lower-limb) coordination, such unique features have provided trainees an opportunity to decelerate the progress of cognitive decline and strengthen muscle-related functions (Zou et al., 2019b; Chen et al., 2020). Tai Chi practice could improve postural control and reduce fall risk in older women (Sun et al., 2018, 2019). Song et al.’s finding indicated that Tai Chi practice improved physical stability during the performance of dual tasks in older adults (Song et al., 2018). In comparison with other exercise interventions, Tai Chi might be a superior strategy for fall reduction through benefitting the cognitive function (Nyman, 2021) and neuromuscular function improvements (Hu et al., 2021). Yuan Yang et al. randomly assigned 26 healthy older women to the Tai Chi group and Control group, with Tai Chi group performing Tai Chi exercise for 8 weeks and Control group engaging in daily activities, and after Tai Chi intervention, the Tai Chi group demonstrated elevated HbO_2_ concentration in the PFC (Yang et al., 2019). Long-term Tai Chi practice improved white matter and delayed the degeneration of brain tissues (Yao et al., 2019).

Regular Tai Chi practice can improve physical postural control ability (Li et al., 2012; Zhou et al., 2015) and cognitive function of older adults to reduce falls (Wu et al., 2013; Zou et al., 2019c). Many previous studies have investigated the effects of Tai Chi on postural control and cognitive function in older adults; however, the researches on the associated brain mechanisms are still scarce. To better understand the role of the PFC in complex walk task and the intervention effect from Tai Chi, we designed a randomized controlled trial with a 16-week Tai Chi intervention, and collected hemodynamic data of the PFC pre- and post-intervention by means of fNIRS. We hypothesized that older adults had higher PFC activation level and better gait performance during negotiating obstacle after Tai Chi practice.

## Materials and methods

### Participants

Participants were recruited from community-dwellers nearby Shandong Sport University. The inclusion criteria were 65–75 years old; a Mini-Mental State Examination score ≥ 24; without cardiovascular, respiratory, musculoskeletal or neurological diseases; able to live independently and perform daily activities; without any regular exercise in the last 2 years, including Tai Chi; and right-handedness. The exclusion criteria were visual impairment and the use of any medication affecting physical balance and nervous system, such as sleeping pills and tranquillizers, in the last 6 months.

The sample size estimation was performed on G*Power software (Germany) based on a randomized controlled trial (Yang et al., 2019) with a significant increase in HbO_2_ of the PFC during executive function assessment in older adults after Tai Chi intervention (Tai Chi group: 0.116 ± 0.143 mmol/l, Control group: −0.003 ± 0.071 mmol/l, *p* < 0.05). By setting significance level to 0.05 and statistical power to 0.85, the effect size and estimated required sample size were calculated to be 0.57 and 24, respectively.

Overall, thirty-six healthy older adults participated in this study and were randomly divided into two groups with 18 people in each group. Thirty-six different random numbers were assigned to all of the participants. Participants with numbers 1–18 were assigned to the Tai Chi group, and the rest were assigned to the Control group. Male participants were assigned to different groups after female participants were assigned to groups to create a similar gender ratio in each group to minimize gender bias. After group assignment, the age and cognitive performance were compared with independent-t tests to ensure no assignment bias. Twenty-seven participants accomplished the intervention, 12 in the Tai Chi group and 15 in the Control group (See details in Table 1). All methods were performed in accordance with the latest guidelines and regulations of the Declaration of Helsinki. This study was approved by the Ethics Review Committee of Shandong Sport University. All participants signed an informed consent form and were told what to do regarding the study.

**Table 1:** Participants’ withdrawal in the Tai Chi group and Control group.

Group	Initial number	Withdrawal number	Reason for withdrawal	Final number
Tai Chi group	18	3	Lost contact	12
		1	For personal reasons	
		2	Withdrew owing to insufficient participation	
Control group	18	3	For personal reasons	15

### Study protocol

Participants in the Tai Chi group practiced Tai Chi for 16 weeks (Kim et al., 2016) four times per week, and 60 min once. Each session was composed of 10 min warm-up, 45 min Tai Chi practice and 5 min cool-down. A certified Tai Chi instructor guided the participants to perform the Tai Chi movements. If the participation rate was less than 80%, the participant was excluded. The participants in the Control group received health lectures for 16 weeks, four times per week, and 60 min once. Both groups maintained their daily living habits.

An 8-form simplified version adapted from the 24-form version of Yang style-based Tai Chi was practiced in this study. The movements included Move a Ball, Part Wild Horse’s Mane, Single Whip, Wave Hands like Clouds, Repulse Monkey, Brush Knees, Fair Lady Works at Shuttle, and Grasp Peacock’s Tail (Li, 2014).

The experiment was conducted at the Sports Biomechanics Laboratory of Shandong Sport University. The participants performed the NOT and negotiating obstacle with the cognitive task (NOCT) at their preferred speed along a 6 m walkway. The obstacle consisted of two upright stands with an unfixed crossbar and was positioned in the midpoint of the walkway. If the participant touched the crossbar, it dropped out to prevent tripping over. The obstacle had the length of 60 cm, the width of 3 cm and the height set to 20% of the participant’s leg length (Zhang et al., 2011; Pan et al., 2016) which was determined by the distance between the anterior superior iliac spine and the medial ankle. The start position was adjusted to ensure comfortably negotiating the obstacle with a minimum of 2 steps before the crossing stride. Several practice trials were instructed to avoid contacting the crossbar and to reduce unnecessary movements contributing to optode shift.

Each test was performed at least three times, including a 30-s rest state and a 30-s walk state. During the rest state, the participant was instructed to sit quietly with eyes closed and stay relaxed. The cognitive task was serial subtraction by 3 from a random three-digit number, involving working memory (Maylor and Wing, 1996). Each participant sat and conducted the cognitive task three times (30 s once) to assess the executive function before the experiment. At the beginning, the researcher verbally provided a three-digit number, and the participant reported the calculation results until the task termination. The walk route was back and forth twice with negotiating obstacle four times in a straight line. The task order was random.

### Apparatus and data acquisition

#### Walking speed

A three-dimensional motion capture system (Vicon, Oxford Metrics Ltd., Oxford, United Kingdom) with 12 infrared cameras (acquisition frequency 100 Hz) was used in this study. Thirty-nine infrared reflective balls (diameter 14 mm) were stuck on the participant’s key bone marker for gait data collection. These data were named, modelled, and intercepted by the data acquisition software (Vicon Nexus 1.7.1) and imported into the Visual 3D software (C-Motion, United States) for modeling, smoothing, and normalization. The fifth lumbar marker was selected as the reference point to calculate the average walking speed of one gait cycle during negotiating obstacle.

#### Brain hemodynamics

**Figure 1:**
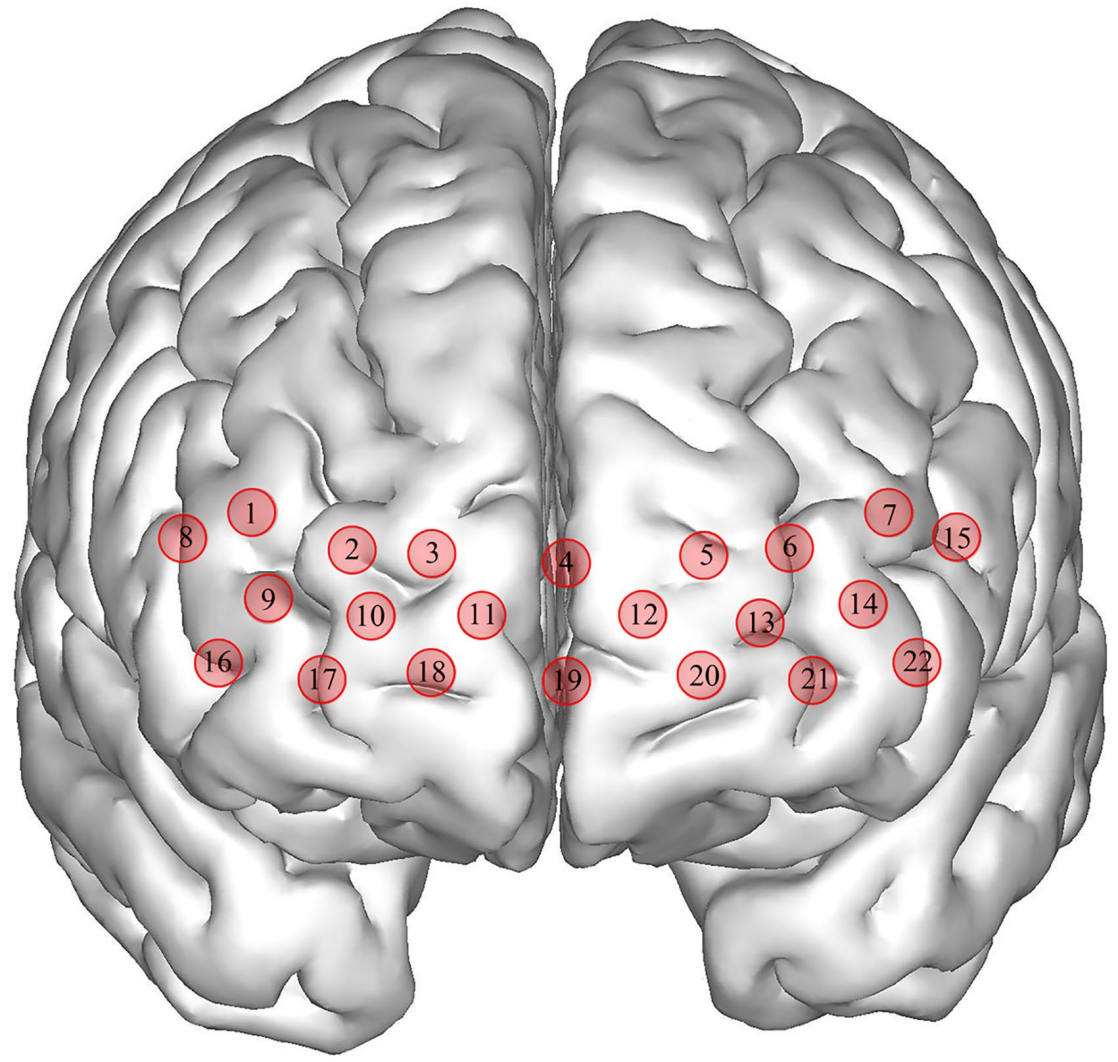
Schematic diagram of channel distribution in fNIRS (front view).

Using a portable fNIRS (LIGHTNIRS, Shimadzu Corp., Kyoto, Japan), the hemodynamic response was measured (BA10 as ROI). The fNIRS had 16 optodes (8 emitters and 8 detectors, sampling frequency13.3 Hz) with 22 channels, and laser diodes (wavelengths 780, 805, and 830 nm). The distance between the adjacent optods was 3 cm. Using a 3D Digitizer (FASTRAK, Polhemus, Vermont), the Montreal Neurological Institute coordinates of channels were determined (See Figure 1) and provided in the supplementary material. During the task test, participants carried the fNIRS in their backpacks while walking and wore a whole-head fiber holder with the standard head landmarks determined according to the international 10/10 system. The lowest row of optodes touched the eyebrows and the line between the nasion and inion passed through the center of channel 19 and 4. We checked each optode and separated the hair beneath it to ensure the probe fully contacted with the scalp. All the optodes were covered with a black cloth to avoid light contamination. The right BA10 included channels 2, 3, 4, 9, 10, and 11, and the left BA10 included channels 4, 5, 6, 12, 13, 14, and 22.

According to the modified Beer–Lambert law, blood oxygen concentrations were obtained, including oxyhemoglobin (HbO_2_), deoxyhemoglobin (HbR), and total hemoglobin (HbT). The visual inspection was performed for the raw hemodynamic signals and channels with a prominent bias or technical artifacts were interpolated by adjacent channels. The episodes irrelevant to the tasks were removed. By the MATLAB-based NIRS_SPM toolkit, the data were processed. The wavelet minimum description length algorithm was applied to remove artifacts caused by respiration, heartbeat, vasodilation, contraction, or other errors related to movement, and to eliminate linear drift. High-frequency noise was reduced/removed by a low-pass filter (cut-off frequency 0.15 Hz) based on the canonical hemodynamic response function (Hiura et al., 2018). The average blood oxygen concentrations within the last 5 s of rest state was used for baseline correction to obtain the change values (ΔHbO_2_, ΔHbR, ΔHbT). The blood oxygen concentration change values in three trials for each task were superimposing averaged to obtain time-series changes of all channels. The channel-wise average data were averaged across all channels in right BA10 and left BA10 respectively, subsequently across all participants. By the Correlation based signal improvement (CBSI) method (Cui et al., 2010), the ΔHbO_2_ and ΔHbR were corrected for the correlation analysis to assess the cortical activation.

#### Dual-task score under NOCT

Dual-task score was calculated to evaluate the cognitive performance according to the following equation:

Dual-task score = correct number/total number (correct number + wrong number)/duration (Mirelman et al., 2017).

#### Dual-task cost

We defined the dual-task cost on walking speed according to the following equation:

Dual-task cost = (single-task walking speed − dual-task walking speed)/single-task walking speed × 100 (Mori et al., 2018).

In general, bigger values indicated a more conservative gait pattern and greater interference by the cognitive tasks.

### Statistical analysis

Statistical analysis was performed using the SPSS 19.0 software. We used the Shapiro–Wilk test to analyze the normality of the data and the Leven’s test to measure the homogeneity of variance. If in accordance with normal distribution, a two-way analysis of variance (ANOVA) with repeated measures was used, age as a covariate, group (Tai Chi group and Control group) as the between-subject factor, and time (pre-intervention and post-intervention) as the within-subject factor. To explore the correlation among PFC activity, walking speed, dual-task score and dual-task cost, Pearson’s correlation was performed (strong:|*r*| ≥ 0.7, moderate: 0.5 ≤ |*r*| < 0.7, weak: 0.3 ≤ |*r*| < 0.5). Means and standard errors were calculated. The significant level was set to 0.05. The Bonferroni method was used for multiple comparison corrections. The effect sizes were expressed with partial Eta squared (*η*_p_^2^), *η*_p_^2^ < 0.06: small effect size, 0.06 < *η*_p_^2^ < 0.14: medium effect size, *η*_p_^2^ *>* 0.14: strong effect size. Cortical activation pictures were visualized with BrainNet Viewer.

## Results

### Demographic data

Before the experiment, demographic variables were analyzed for both groups (Table 2). No significant differences in age, height, weight and cognitive performance were observed between the groups (*p* > 0.05).

**Table 2:** Participants’ demographic data in the Tai Chi group and Control group.

Characteristics	Tai Chi group	Control group	*t*	*p*
Age (year)	67.67 ± 0.68	70.13 ± 1.06	1.928	0.066
Height (cm)	164.33 ± 2.14	162.53 ± 1.19	−0.712	0.486
Weight (kg)	63.19 ± 2.63	65.01 ± 2.62	0.477	0.638
Cognitive performance(/s)	0.026 ± 0.006	0.023 ± 0.001	−1.369	0.183

### Comparison of walking speed pre- and post-intervention

**Figure 2:**
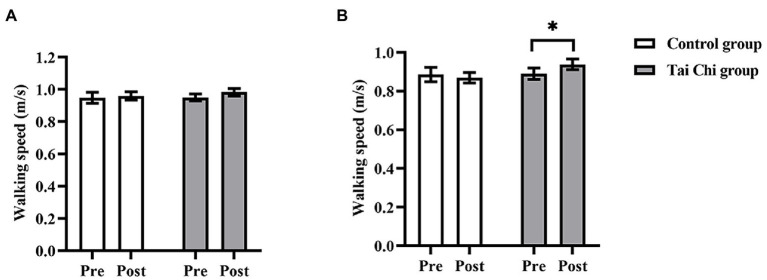
Walking speed in the Tai Chi group and Control group under NOT **(A)** and NOCT **(B)** pre- and post- intervention. *Represents significant difference (*p* < 0.05).

Two-way ANOVA with repeated measures was used to analyze walking speed and an interaction effect of group and time was observed under NOCT, but not under NOT (NOCT: *F* = 4.582, *p* = 0.043, *η*_p_^2^ = 0.160; NOT: *F* = 0.440, *p* = 0.513, *η*_p_^2^ = 0.018). Before the intervention, no significant differences in walking speed were observed between both groups under NOT and NOCT, and participants were at the same baseline level (NOCT: *F* = 0.038, *p* = 0.848, η_p_^2^ = 0.002; NOT: *F* = 0.001, *p* = 0.969, *η*_p_^2^ < 0.001). Walking speed post-intervention was significantly faster than pre-intervention in the Tai Chi group under NOCT (*F* = 4.708, *p* = 0.040, *η*_p_^2^ = 0.164) and faster than in the Control group without statistical difference (*F* = 3.160, *p* = 0.088, *η*_p_^2^ = 0.116; Figure 2). Thus, Tai Chi intervention improved walking speed performance of the older adults when negotiating obstacle under a dual-task condition.

### Comparison of dual-task cost pre- and post-intervention

**Figure 3:**
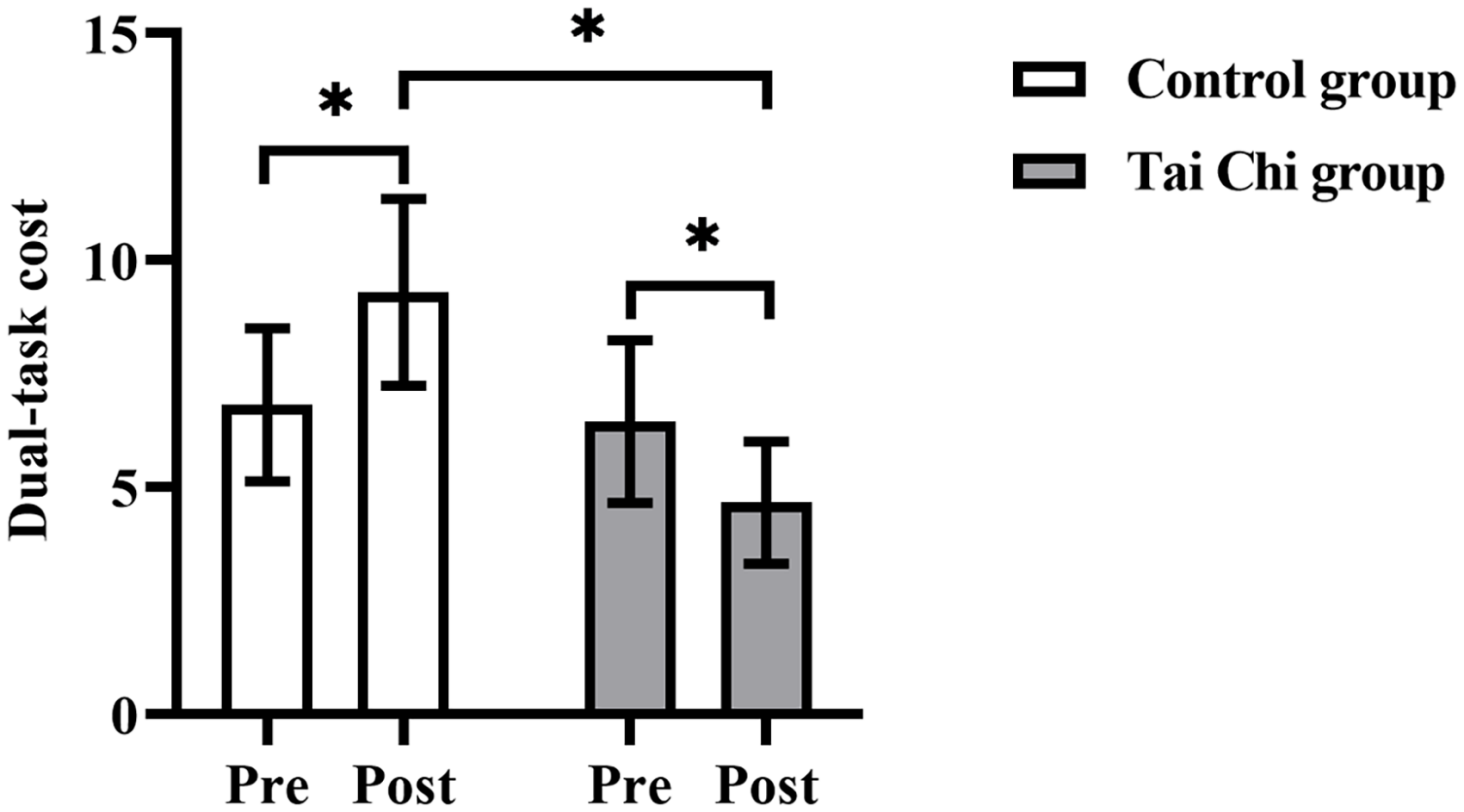
Dual-task cost in the Tai Chi group and Control group pre- and post- intervention. *Represents significant difference (*p* < 0.05).

Two-way ANOVA with repeated measures was used to analyze dual-task cost and an interaction effect of group and time was observed (*F* = 5.067, *p* = 0.034, *η*_p_^2^ = 0.174). *Post hoc* test indicated dual-task cost post-intervention were significantly lower in the Tai Chi group (*F* = 4.393, *p* = 0.047, *η*_p_^2^ = 0.155) and significantly higher in the Control group (*F* = 4.248, *p* = 0.050, *η*_p_^2^ = 0.150), compared to pre-intervention (Figure 3). After the intervention, dual-task cost was significantly lower in the Tai Chi group than in the Control group (*F* = 5.232, *p* = 0.037, *η*_p_^2^ = 0.174). No significant difference between two groups at pre-intervention (*F* = 0.294, *p* = 0.593, *η*_p_^2^ = 0.012), indicating the two groups were at the same baseline level.

### Comparison of blood oxygen concentration changes pre- and post-intervention

**Figure 4:**
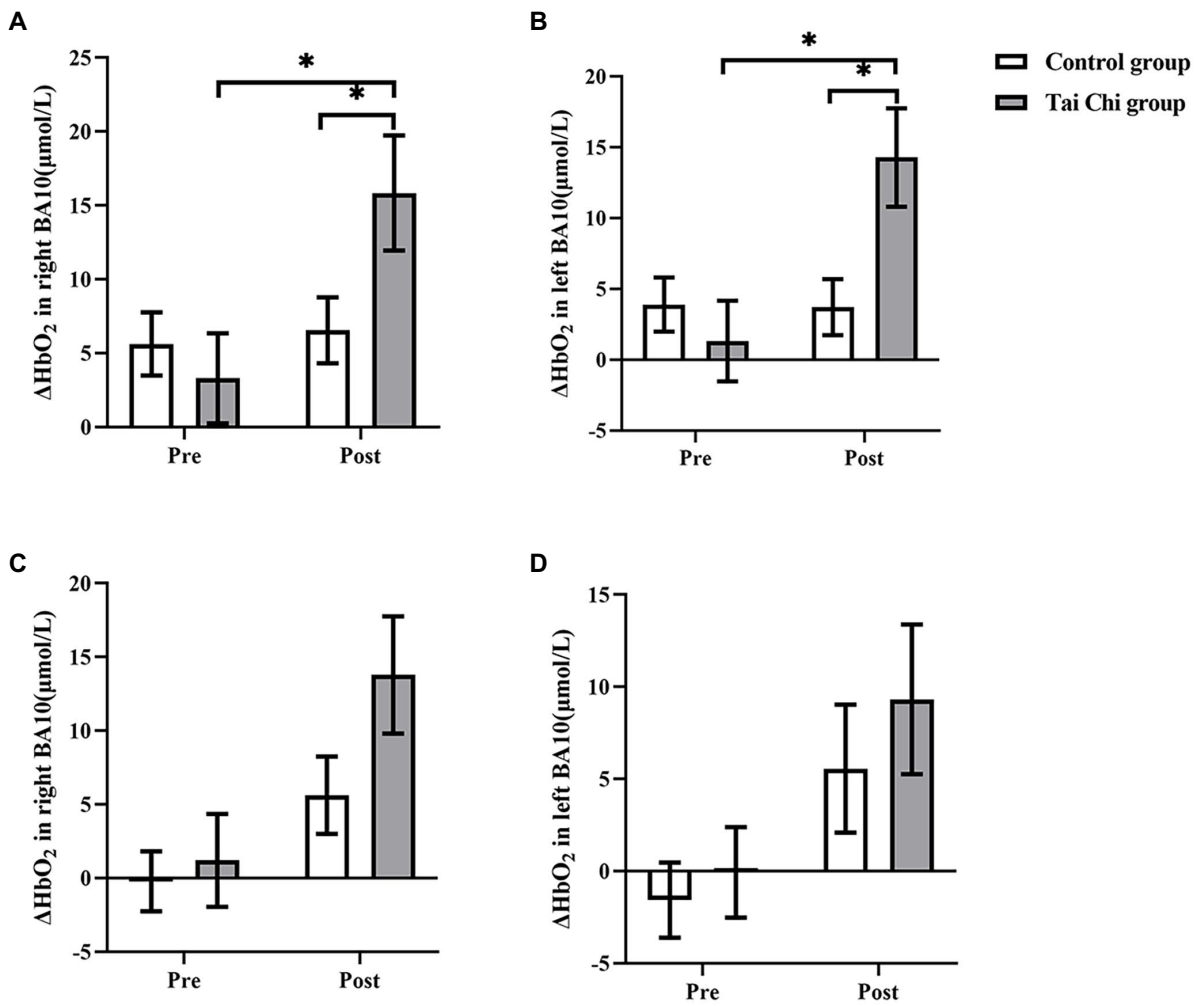
ΔHbO_2_ in BA10 in the Tai Chi group and Control group under NOCT and NOT pre- and post-intervention. **(A–D)** represent right BA10 under NOCT, left BA10 under NOCT, right BA10 under NOT, and left BA10 under NOT respectively. *Represents significant difference (*p* < 0.05).

**Figure 5:**
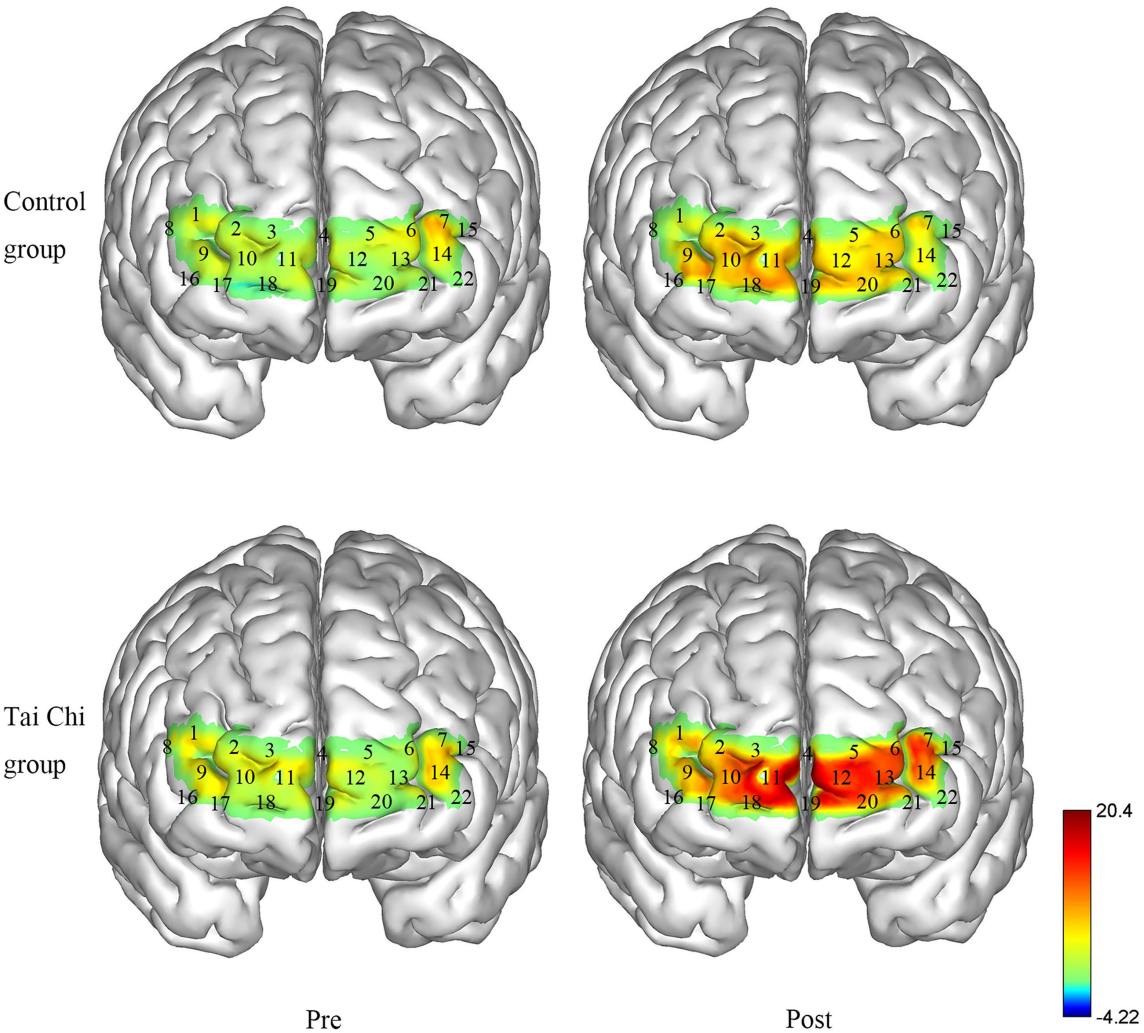
Changes in the ΔHbO_2_ in the Tai Chi group and Control group under NOCT pre- and post-intervention (μmol/L). The right BA10 includes channels 2, 3, 4, 9, 10, and 11, and the left BA10 includes channels 4, 5, 6, 12, 13, 14, and 22.

**Figure 6:**
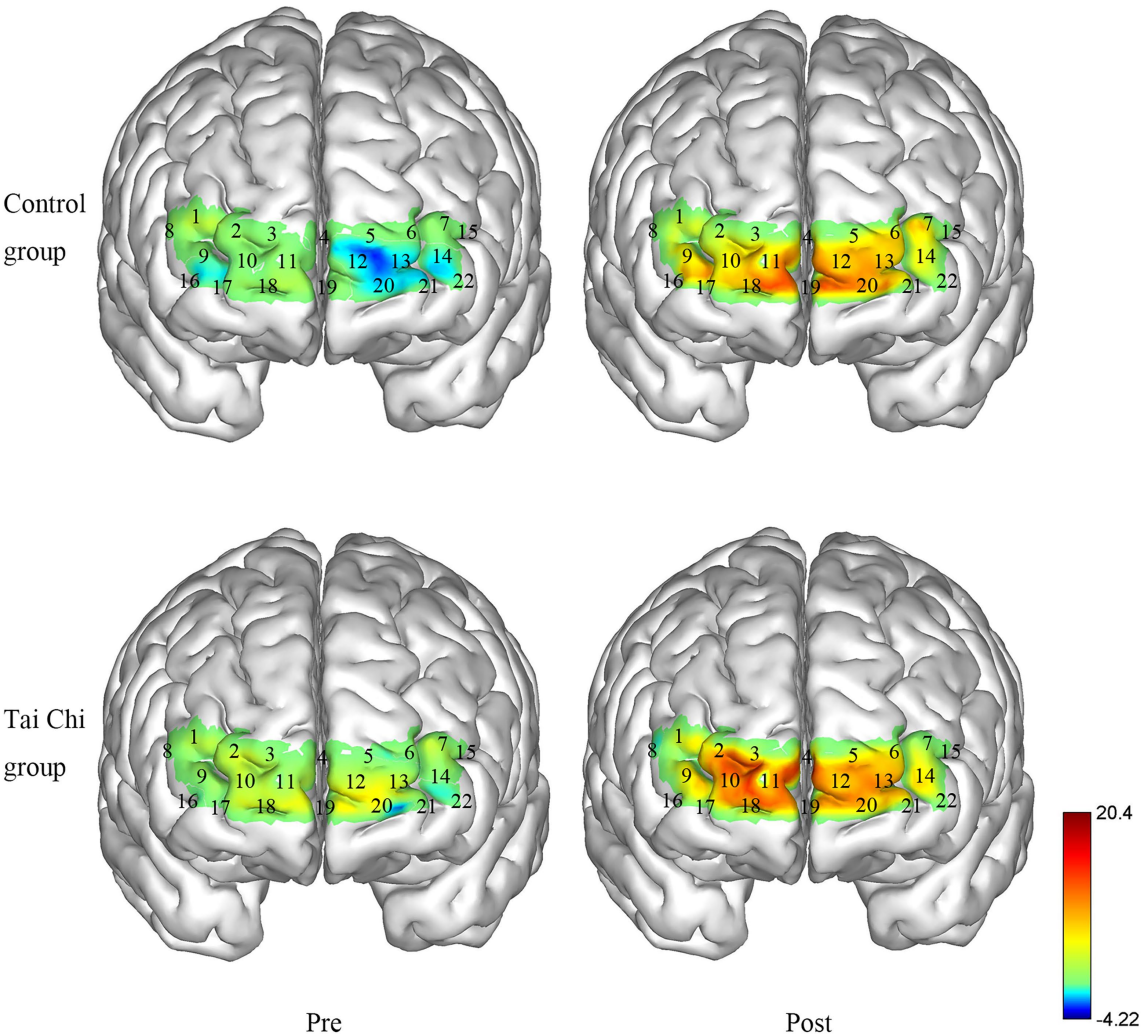
Changes in the ΔHbO_2_ in the Tai Chi group and Control group under NOT pre- and post-intervention (μmol/L). The right BA10 includes channels 2, 3, 4, 9, 10, and 11, and the left BA10 includes channels 4, 5, 6, 12, 13, 14, and 22.

Two-way ANOVA with repeated measures was applied for ΔHbO_2_ in right BA10 and left BA10. An interaction effect of group and time was identified under NOCT (right: *F* = 5.332, *p* = 0.030, *η*_p_^2^ = 0.182; left: *F* = 8.901, *p* = 0.006, *η*_p_^2^ = 0.271). *Post hoc* test indicated ΔHbO_2_ in BA10 were significantly higher after Tai Chi intervention (right: *F* = 11.404, *p* = 0.002, *η*_p_^2^ = 0.322; left: *F* = 15.851, *p* = 0.001, *η*_p_^2^ = 0.398), and significantly higher in the Tai Chi group compared to the Control group (right: *F* = 4.287, *p* = 0.049, *η*_p_^2^ = 0.153; left: *F* = 6.384, *p* = 0.019, *η*_p_^2^ = 0.210; Figures 4, 5). No significant difference between two groups pre-intervention (right: *F* = 0.947, *p* = 0.340, *η*_p_^2^ = 0.038; left: *F* = 0.574, *p* = 0.456, *η*_p_^2^ = 0.023) indicated the two groups were at the same baseline level. Under NOT, there was no interaction effect of group and time (right: *F* = 0.181, *p* = 0.674, *η*_p_^2^ = 0.007; left: *F* = 0.063, *p* = 0.803, *η*_p_^2^ = 0.003). There was the main effect of time but not of group in right BA10 (group: *F* = 0.628, *p* = 0.436, *η*_p_^2^ = 0.026; time *F* = 13.044, *p* = 0.001, *η*_p_^2^ = 0.352). There were not the main effects of group and time in left BA10 (group *F* = 1.426, *p* = 0.244, *η*_p_^2^ = 0.054; time *F* = 2.879, *p* = 0.102, *η*_p_^2^ = 0.103; Figures 4, 6).

**Figure 7:**
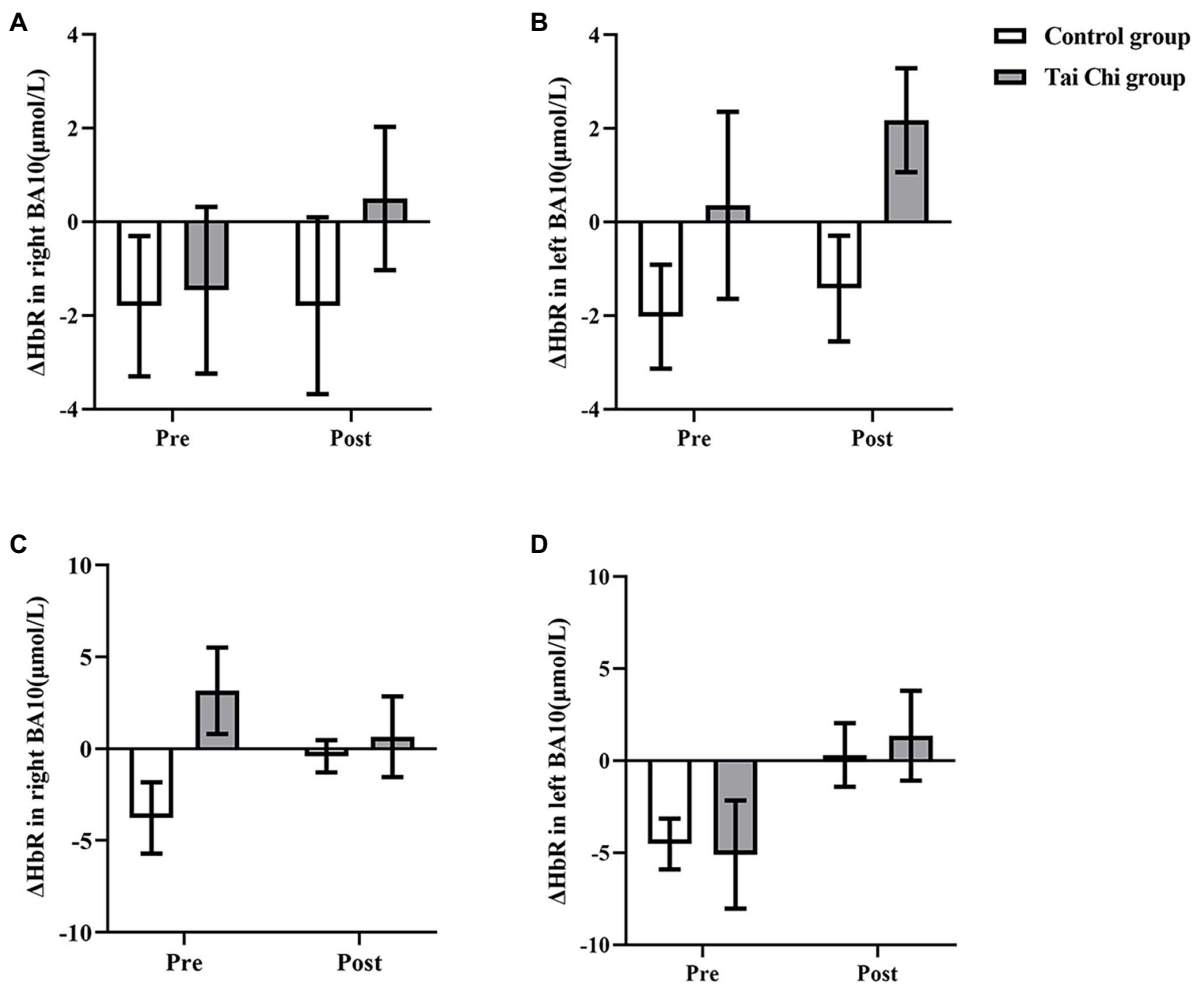
ΔHbR in BA10 in the Tai Chi group and Control group under NOCT and NOT pre- and post-intervention. **(A–D)** represent right BA10 under NOCT, left BA10 under NOCT, right BA10 under NOT, and left BA10 under NOT respectively. *Represents significant difference (*p* < 0.05).

Two-way ANOVA with repeated measures was applied for ΔHbR in right BA10 and left BA10. No group and time interactions were found under NOT and NOCT (NOT: right: *F* = 0.029, *p* = 0.867, *η*_p_^2^ = 0.001; left: *F* = 0.028, *p* = 0.868, *η*_p_^2^ = 0.001; NOCT: right: *F* = 0.322, *p* = 0.576, *η*_p_^2^ = 0.013; Left: *F* = 0.447, *p* = 0.510, *η*_p_^2^ = 0.018; Figure 7). The main effects of group and time in right BA10 were not statistically significant under NOT and NOCT (NOT: group: *F* < 0.001, *p* = 0.990, *η*_p_^2^ < 0.001; time: *F* = 3.146, *p* = 0.089, *η*_p_^2^ = 0.116; NOCT: group: *F* = 0.826, *p* = 0.372, *η*_p_^2^ = 0.033; time: *F* = 0.308, *p* = 0.584, *η*_p_^2^ = 0.013). The main effect of group in left BA10 was not statistically significant, while the main effect of time was statistically significant under NOT (group: *F* = 0.010, *p* = 0.920, *η*_p_^2^ < 0.001; time: *F* = 5.159, *p* = 0.032, *η*_p_^2^ = 0.177). The main effect of group in left BA10 was statistically significant, while the main effect of time was not statistically significant under NOCT (group: *F* = 4.279, p = 0. 050, *η*_p_^2^ = 0.151; time: *F* = 0.921, *p* = 0.347, *η*_p_^2^ = 0.037).

**Figure 8:**
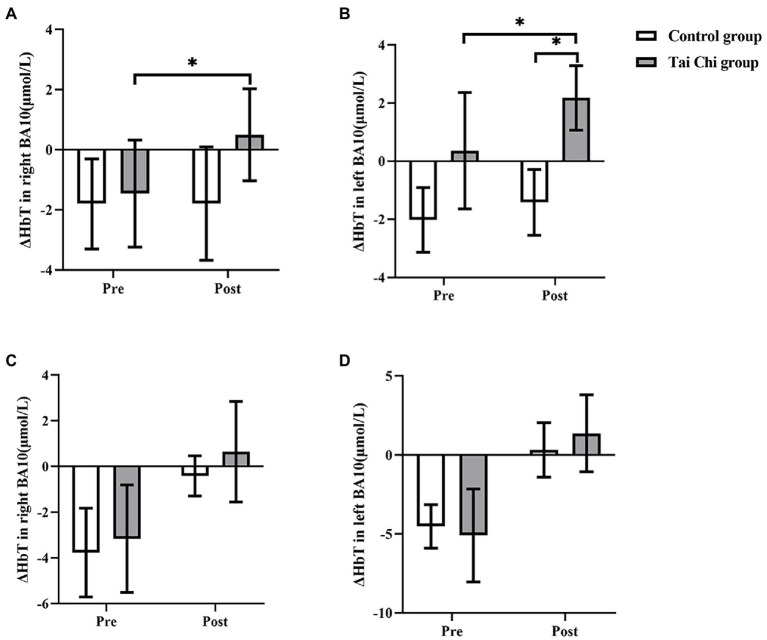
ΔHbT in BA10 in the Tai Chi group and Control group under NOCT and NOT pre- and post-intervention. **(A–D)** represent right BA10 under NOCT, left BA10 under NOCT, right BA10 under NOT, and left BA10 under NOT respectively. *Represents significant difference (*p* < 0.05).

Two-way ANOVA with repeated measures was applied for ΔHbT in right BA10 and left BA10. An interaction effect of group and time was identified under NOCT (right: *F* = 6.117, *p* = 0.021, *η*_p_^2^ = 0.203; left: *F* = 7.274, *p* = 0.013, *η*_p_^2^ = 0.233). *Post hoc* test indicated ΔHbT in BA10 was significantly higher after Tai Chi intervention (right: *F* = 9.090, *p* = 0.006, *η*_p_^2^ = 0.275; left: *F* = 11.213, *p* = 0.003, *η*_p_^2^ = 0.318). ΔHbT in left BA10 was significantly higher whereas ΔHbT in right BA10 was higher without statistical difference in the Tai Chi group compared to the Control group (right: *F* = 4.081, *p* = 0.055, *η*_p_^2^ = 0.145; left: *F* = 7.796, *p* = 0.010, *η*_p_^2^ = 0.245; Figure 8). No significant differences between two groups at pre-intervention (right: *F* = 0.668, *p* = 0.422, *η*_p_^2^ = 0.027; left: F = 0.028, *p* = 0.869, *η*_p_^2^ = 0.001) indicated two groups were at the same baseline level. Under NOT, there was no interaction effect of group and time (right: *F* = 0.090, *p* = 0.767, *η*_p_^2^ = 0.004; left: *F* = 0.017, *p* = 0.899, *η*_p_^2^ = 0.001). The main effect of group was not statistically significant (right: *F* = 0.289, *p* = 0.596, *η*_p_^2^ = 0.0126; left: *F* = 0.161, *p* = 0.692, *η*_p_^2^ = 0.007). The main effect of time was statistically significant (right: *F* = 10.200, *p* = 0.004, *η*_p_^2^ = 0.298; left: *F* = 15.647, *p* = 0.001, *η*_p_^2^ = 0.395).

### Dual-task score under NOCT

The statistical analysis results revealed no interaction effects in group and time (*F* = 0.587, *p* = 0.451, *η*_p_^2^ = 0.024). No significant differences were observed in dual-task score pre-intervention between two groups (F = 0.587, *p* = 0.451, *η*_p_^2^ = 0.024). The main effects of group and time were not statistically significant (group: *F* = 0.738, *p* = 0. 399, *η*_p_^2^ = 0.030; time: *F* = 0.770, *p* = 0.389, *η*_p_^2^ = 0.031).

### Correlation among PFC ΔHbO_2_, dual-task score, dual-task cost and walking speed under NOCT

Before the intervention under NOCT, ΔHbO_2_ in left BA10 was negatively correlated with dual-task cost (*p* = 0.001). Whereas ΔHbO_2_ in right BA10 was not correlated with dual-task cost (*p* = 0.199) and walking speed (*p* = 0.287). After the intervention under NOCT, ΔHbO_2_ in left BA10 was negatively correlated with dual-task cost (*p* = 0.019) and positively correlated with walking speed (*p* = 0.016). ΔHbO_2_ in right BA10 was negatively correlated with dual-task cost (*p* = 0.021) and positively correlated with walking speed (*p* = 0.015; see the details in Table 3).

**Table 3:** Pearson’s correlation analysis among ΔHbO_2_, walking speed, dual-task cost and dual-task score under NOCT.

		Walking speed		Dual-task cost		Dual-task score	
		*r*	*p*	*r*	*p*	*r*	*p*
Pre	ΔHbO_2_ in right BA10	0.213	0.287	−0.255	0.199	0.176	0.381
	ΔHbO_2_ in left BA10	0.290	0.143	−0.596	**0.001**	0.256	0.198
	Walking speed			−0.588	**0.001**	0.125	0.535
	Dual-task cost					−0.181	0.366
Post	ΔHbO_2_ in right BA10	0.461	**0.015**	−0.443	**0.021**	0.077	0.704
	ΔHbO_2_ in left BA10	0.458	**0.016**	−0.448	**0.019**	0.227	0.255
	Walking speed			−0.595	**0.001**	0.029	0.886
	Dual-task cost					−0.010	0.961

Pearson's correlation coefficient (*r*) and *p*-value, the significant level was highlighted by bold font.

Dual-task cost was negatively correlated with walking speed under NOCT (pre-intervention: *p* = 0.001; post-intervention: *p* = 0.001), whereas dual-task scores were not correlated with ΔHbO_2_, dual-task cost and walking speed (*p* > 0.05), either pre- or post-intervention (see the details in Table 3).

After the CBSI correction, there were strong negative correlations between ΔHbO_2_ and ΔHbR under NOCT, either pre-intervention (left PFC *r* = −0.841, *p* < 0.001; right PFC *r* = −0.795, *p* < 0.001) or post-intervention (left PFC *r* = −0.842, *p* < 0.001; right PFC *r* = −0.744, *p* < 0.001). There were strong/moderate negative correlations between ΔHbO_2_ and ΔHbR under NOT, either pre-intervention (left PFC *r* = −0.739, *p* < 0.001; right PFC *r* = −0.690, *p* < 0.001) or post-intervention (left PFC *r* = −0.782, *p* < 0.001; right PFC *r* = −0.540, *p* = 0.004).

## Discussion

In this study, we investigated the effects of a 16-week Tai Chi intervention on cortical activation and gait performance of older adults during negotiating obstacle in single-task and dual-task conditions. After Tai Chi intervention under NOCT, ΔHbO_2_ with a significant increase and ΔHbR with no significant change or slight increase, which was probably induced by head motion in the more naturalistic experiment (Cui et al., 2010) i.e., walking and negotiating obstacles. After the CBSI correction, there were strong negative correlations between ΔHbO_2_ and ΔHbR under NOCT either pre-intervention or post-intervention. The research results revealed: (1) after Tai Chi intervention, PFC activation was bilateral with lower dual-task cost under NOCT, meanwhile these were not observed under NOT; (2) PFC ΔHbO_2_ under NOCT was negatively correlated with dual-task cost and not correlated with dual-task score.

With aging, walking was less automated and required more attentional resources. When walking became more difficult under a dual-task condition, PFC showed higher activation (Mirelman et al., 2017). In the Tai Chi group, when older adults performed cognitive task and negotiating obstacle simultaneously, the PFC was bilaterally activated and compensated for the cognitive resource deficit. Our findings were in line with the revised scaffolding theory of aging and cognition, suggesting that the older adults may adjust to age-related neurodegeneration through recruiting additional neural networks (Reuter-Lorenz and Park, 2014). Increased PFC activation was an indicator of brain adaption with compensation to maintain the performance as a result of declining neural functions and structures (Park and Reuter-Lorenz, 2009). After Tai Chi intervention, lower dual-task cost exhibited a more active gait pattern and less interference from cognitive task. PFC activation under NOCT was negatively correlated with dual-task cost. The high-level prefrontal activity was linked to optimizing gait performance during a complex walking task, such as negotiating obstacle. When encountering an obstacle (Ohsugi et al., 2013; Clark et al., 2014), successful gait adaptability was crucial and associated with processing speed for precise foot placements and cognitive capacity for step length adjustments (Caetano et al., 2017). Impaired gait adaptability contributed to high risk of falls in older adults and reduced executive function significantly enhanced the link (Caetano et al., 2016, 2018). Healthy older adults required additional PFC resources for task demands, and this overactivation might lead to better external performance (Albinet et al., 2014; Dupuy et al., 2015; Hyodo et al., 2016). For older adults, Tai Chi practice possibly enabled higher PFC activation and better gait performance to cope with challenges during walking.

PFC activation under NOCT was negatively correlated with dual-task cost, and not correlated with dual-task score. PFC activation might allocate more attention to motor ability but not calculation task, which was not consistent with that of Takayuki Mori et al. (2018). Their findings indicated that healthy participants prioritized the cognitive task during dual-task walking. One possible explanation for the difference was a greater age range (range 30–74 years) in contrast with this study (range 65–75 years). Some studies have suggested that young and older adults spontaneously prioritized gait stability during walking if no specific prioritization instruction or allocation of attention was provided, this strategy by which they tended to prioritize motor ability was known as ‘posture first’(Shumway-Cook et al., 1997). Moreover, when given the choice between a cognitive or motor performance aid, older adults selected the option to supplement gait performance (Li et al., 2001), and this postural prioritization strategy helped to prevent the loss of balance (Shumway-Cook et al., 1997; Li et al., 2001; Yogev-Seligmann et al., 2010; Chen et al., 2022). Interestingly, after Tai Chi intervention, walking speed was not faster, and PFC activation was not greater under NOT. This finding might indicate the available cognitive resources were sufficient for maintaining gait performance during negotiating obstacle, and kept resource-saving in older adults, although Tai Chi practice increased the capacity of available resources.

Originating from martial arts, Tai Chi was a mind–body exercise that integrated flexibility and coordination, with performance driven by an inner to outer process (i.e., mind-initiated and directed inside, movement synergies outside; Li, 2014; Yang et al., 2019). Tai Chi practice required not only simple to complex, balance-challenging, multi-joint directed postural control movements, but also memory training for complex movement sequences and associated rhythms, as well as various higher cognitive functions (e.g., visuospatial ability, attention, multitask planning, and processing) to maintain postural stability and orientation. During negotiating obstacle, the visuospatial ability was required to load all information about the obstacle (such as the distance to the obstacle, and the size of the obstacle), and Tai Chi focused on visuospatial training. When physical-spatial orientations and action directions were frequently inconsistent during Tai Chi practice, older adults needed to deal with this conflicting information and select a proper action, which required specific cognitive activities, including motion recall and task switching (Yang et al., 2019). Long-term Tai Chi practice could lead to changes in the local brain structures (Wei et al., 2014) and enhance functional connectivity (Tao et al., 2016, 2017). In a study analyzing the brain structures of older adults with long-term Tai Chi practice, the frontal middle sulcus was significantly thicker, suggesting that Tai Chi practice could cause structural changes in the PFC (Wei et al., 2013). The frontal middle sulcus was located in the dorsolateral PFC and partially overlapped with BA10 (the ROI in this study), which provided the evidence that Tai Chi practice changed the local structures of the PFC and improved executive function. The HAROLD model (hemispheric asymmetry reduction in older adults) stated the prefrontal activity was inclined to manifest less lateralization in older adults than in younger adults, attributing to a compensatory function or a dedifferentiation process (Cabeza, 2002). From a computational benefit hypothesis, the functional lateralization could be known as a special case of the functional specialization of the brain (Kosslyn, 1987) to process the information more efficiently. The age-related hemispheric asymmetry reduction reflected the decrease in neural processing capacity by performing simultaneous processing (Rogers et al., 2004). After Tai Chi intervention, the bilateral activation mode could help offset age-related neurocognitive deficits, which could be achieved by alternative neurocognitive networks (Reuter-Lorenz and Lustig, 2005).

This study had some limitations. First, we did not monitor skin blood flow, which might interfere fNIRS signal (Kirilina et al., 2012; Erdogan et al., 2014). Second, no physiological parameters, such as heart rate, and rate of perceived exertion, were measured during Tai Chi intervention, although Tai Chi was considered to be a low to moderate-intensity exercise. Third, the fNIRS equipment had 16 optodes, so that only the PFC was detected, failing to involve other brain regions related to motor control or visual processing, such as the premotor, sensorimotor, and occipital cortices. Fourth, this study recruited participants in a narrow age range from 65 to 75 to avoid the influences of heterogeneity among older adults, which may affect the applicability of our outcomes to other age populations. Fifth, consequence of interindividual response variability should be considered for future studies as these variables are linked to exercise-induced benefits (Herold et al., 2021). Lastly, eye-based measures are recently recommended by a group of experts exercise-cognition field as they can be used in combination with fNIRS, leading to more reliable findings.

## Conclusion

Tai Chi practice might increase cognitive resources of older adults through the PFC bilateral activation to prioritize gait performance during negotiating obstacle under a dual-task condition.

## Data availability statement

The raw data supporting the conclusions of this article will be made available by the authors, without undue reservation.

## Ethics statement

The studies involving human participants were reviewed and approved by the Ethics Review Committee of Shandong Sport University. The patients/participants provided their written informed consent to participate in this study.

## Author contributions

DM: conceptualization and project design. YC: methodology, experiment implementation and drafting manuscript. AW: data acquisition, visualization and software. MM: investigation and experiment implement. WS: data curation and methodology. QS: supervision and funding acquisition. All authors contributed to the article and approved the submitted version.

## Funding

This work was supported by China Shandong Province Youth Innovative Talent Induction Program (grant number 2019-183).

## Conflict of interest

The authors declare that the research was conducted in the absence of any commercial or financial relationships that could be construed as a potential conflict of interest.

## Publisher’s note

All claims expressed in this article are solely those of the authors and do not necessarily represent those of their affiliated organizations, or those of the publisher, the editors and the reviewers. Any product that may be evaluated in this article, or claim that may be made by its manufacturer, is not guaranteed or endorsed by the publisher.
